# Persistence of the Effects of Se-Fertilization in Olive Trees over Time, Monitored with the Cytosolic Ca^2+^ and with the Germination of Pollen

**DOI:** 10.3390/plants10112290

**Published:** 2021-10-25

**Authors:** Alberto Marco Del Pino, Luca Regni, Roberto D’Amato, Alessandro Di Michele, Primo Proietti, Carlo Alberto Palmerini

**Affiliations:** 1 Department of Agricultural, Food and Environmental Sciences (DSA3), University of Perugia, Borgo XX Giugno 74, 06121 Perugia, Italy; alberto.delpino@unipg.it (A.M.D.P.); roberto.damato@unipg.it (R.D.); carlo.palmerini@unipg.it (C.A.P.); 2 Department of Physics and Geology, University of Perugia, Via Pascoli, 06123 Perugia, Italy; alessandro.dimichele@unipg.it

**Keywords:** *Olea europaea* L., selenium biofortification, oxidative stress, olive pollen, cytosolic Ca^2+^

## Abstract

Selenium (Se) is an important micronutrient for living organisms, since it is involved in several physiological and metabolic processes. Biofortification with Se increases the nutritional and qualitative values of foods in Se-deficient regions and increases tolerance to oxidative stress in olive trees. Many studies have shown that Se, in addition to improving the qualitative and nutritional properties of EVO oil, also improves the plant’s response to abiotic stress. This study addressed this issue by monitoring the effects of Se on cytosolic Ca^2+^ and on the germination of olive pollen grains in oxidative stress. The olive trees subjected to treatment with Na-selenate in the field produced pollen with a Se content 6–8 times higher than the controls, even after 20 months from the treatment. Moreover, part of the micronutrient was organic in selenium methionine. The higher selenium content did not produce toxic effects in the pollen, rather it antagonized the undesirable effects of oxidative stress in the parameters under study. The persistence of the beneficial effects of selenium observed over time in pollens, in addition to bringing out an undisputed adaptability of olive trees to the micronutrient, suggested the opportunity to reduce the number of treatments in the field.

## 1. Introduction

Selenium (Se) is an essential microelement normally present in humans and its endogenous levels fluctuate among populations of different geographic areas and are influenced by environmental factors [1,2]. The microelement, despite being used for many years in the prevention of many diseases, is used with caution as a food additive due to its toxicity at high concentrations [1]. Se in inorganic and organic forms is absorbed by the small intestine and distributed to various tissues, and enters as selenium–cysteine and selenium–methionine in proteins and participates in important biological processes [3,4]. The therapeutic role of selenium was identified since 1957 by Wrobel, through the observation that at low doses it can prevent liver necrosis in rats [3]. Subsequently, many studies have showed beneficial effects of selenium in processes such as immuno-endocrine, metabolic processes and in the maintenance of cellular homeostasis [4,5]. Moreover, the Se biofortification is considered as an agronomic-based strategy, utilized by farmers to produce Se-enriched food products which may help reduce dietary deficiencies in Se-deficient regions such as the Mediterranean Basin [6,7,8,9,10,11]. In recent years, the Se biofortification has shown beneficial effects in plants by increasing the antioxidant defense against reactive oxygen species (ROS) in vegetative growth and in the response to environmental stress [12,13,14,15,16]. ROS, normally produced at low concentrations, participate in membrane signals, reproduction and pollen—stigma recognition [17,18,19]. ROS become toxic at high concentrations, induce oxidative stress and deregulate molecular signals including cytosolic Ca^2+^ [20,21,22,23,24]. Several studies conducted on the cytosolic Ca^2+^ of olive pollen, among the molecular signalling networks, resulted in a reliable experimental model [25,26]. The levels of Ca^2+^ are closely related to the cytosolic concentration of the ions, which changes over time are possible to trace through the marking of pollen with the FURA-2AM probe [25,26,27]. Furthermore, the fluctuations of cytosolic Ca^2+^ have an important role in the germination of pollen and in the growth of the pollen tube, even if up to now the interactions between the two events are little known. [25,26]. The objective of this work is to evaluate the half-life of selenium in olive trees and the persistence of the beneficial effects in oxidative stress in order to reduce the number of treatments in the field with Na-selenate. This objective was verified by monitoring the fluctuations of cytosolic Ca^2+^ and the germination rate of olive pollen in oxidative stress.

## 2. Results

### 2.1. Scanning Electron Microscopy Images of Olive Pollen Grains

**Figure 1:**
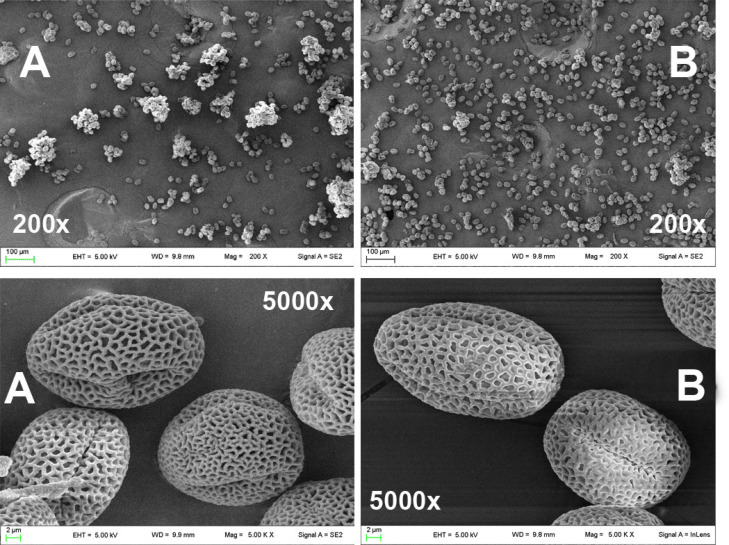
SEM images (200× and 5000×) of olive pollen from control plants (**A**) and from Se-fertilized plants (**B**).

Pollen grains collected after 8 and 20 months from untreated (C_8_, C_20_) and Se-fertilized (T_8_, T_20_) olive trees were analyzed by scanning field emission electron microscopy (SEM). Pollen images from untreated plants (A) and, Se-fertilized (B) are shown in Figure 1. The individual granules of the two populations did not show differences in size and shape, but showed some qualitative differences in morphology: an angular structure instead of a smooth one, a violation of the pattern and thickness of the cuticle (Figure 1). Furthermore, the population of pollen grains from Se-fertilized olive trees (T_8_, T_20_) showed a lower aggregation capacity than that of untreated plants (C_8_, C_20_) in both harvesting periods.

### 2.2. Speciation of Selenium in Olive Pollen

Olive pollen grains collected after 8 and 20 months from untreated (C_8_ and C_20_) and Se-fertilized (T_8_ and T_20_) plants were analyzed by ICPMS HPLC. The analyses showed that the total selenium content (Se-tot.) in the pollens of the Se-fertilized plants was higher than that of the untreated plants (Table 1). 

The ratio [Se-tot. (T_8_/C_8_)] was 8.8 in pollen collected after 8 months and dropped to 5.0 [Se-tot. (T_20_/C_20_)] in the pollen collected after 20 months from the field treatment.

Part of the Na-selenate of the treatment was transformed in selenium–methionine (Se-met) as the predominant species. The Se-met decreased over time and the ratio [Se-met (T_8_)/Se tot. (T_8_)] of 17.2% dropped to 14.2% [Se-met (T_20_)/Se-tot. (T_20_)].

Selenium (VI), the predominant species of inorganic selenium, remained fairly stable. The ratio [Se (VI) (T_8_)/Se-tot. (T_8_)] was 55.9% and increased to 81.3% [Se (VI) (T_20_)/Se-tot. (T_20_)] after 20 months (Table 1).

### 2.3. The Cytosolic Ca^2+^ Tested in Pollen during Oxidative Stress

The labelling of olive pollen grains with the FURA-2AM fluorescent probe allowed to determine the variations over time of cytosolic pollen calcium (Δ[Ca^2+^]_cp_) in oxidative stress. The experiment was conducted in two phases, initially in the absence of Ca^2+^ in the incubation medium, then after 200 s, CaCl_2_ (1 mM) was added. The two phases of the measurement made it possible to differentiate the fluctuations of the cytosolic Ca^2+^ from those deriving from the entry of Ca^2+^ from the extracellular medium.

**Figure 2:**
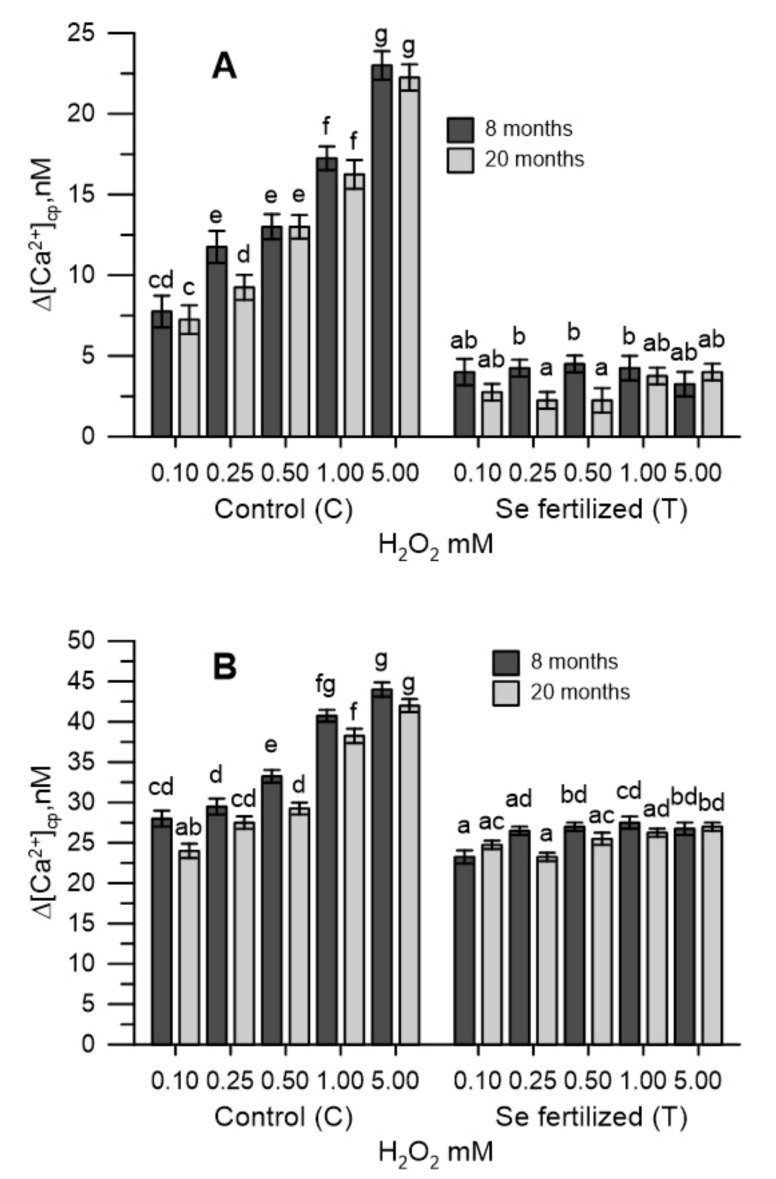
Effect of H_2_O_2_ (0.1 to 5.0 mM) in the [Ca^2+^]_cp_ of pollen grains from untreated (C_8_ and C_20_) and Se-fertilized plants (T_8_ and T_20_). Measurements were performed in the absence (**A**) and in the presence (**B**) of CaCl_2_ (1 mM) in the incubation medium. Data are expressed as means ± SEM from 4 independent tests. Different letters show significant difference at *p* < 0.05.

The [Ca^2+^]_cp_ of pollen from untreated plants (C_8_ and C_20_) was perturbed by oxidative stress induced in vitro with H_2_O_2_ (0.1–5.0 mM), while that of Se-fertilized plants was not. In particular, pollens from untreated trees (C_8_ and C_20_) showed an increase in cytosolic Ca^2+^ proportional to the H_2_O_2_ used, while those from Se-fertilized trees (T_8_ and T_20_) did not show any changes in [Ca^2+^]_cp_ at the same concentrations of hydrogen peroxide (Figure 2A).

The addition of CaCl_2_ (1 mM) determined an increase in [Ca^2+^]_cp_ only in the pollen of untreated plants (C_8_ and C_20_) (Figure 2B).

### 2.4. The Cytosolic Ca^2+^ Tested in Olive Pollen in the Presence of the Extracts of the Germinative Apexes

**Figure 3:**
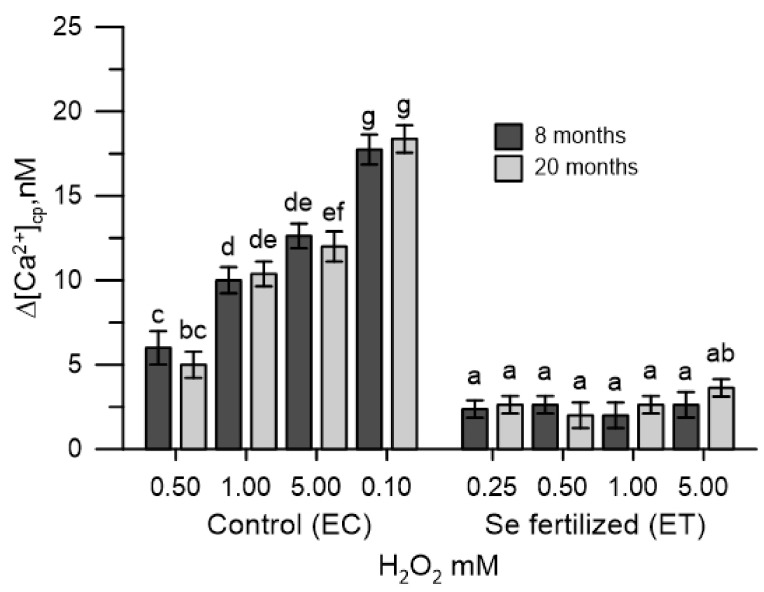
Effects of H_2_O_2_ (0.25 to 5.0 mM) in the cytosolic Ca^2+^ of olive pollen, in the presence of the extracts of the germinative apexes from untreated plants (EC_8_, EC_20_) and Se-fertilized (ET_8_, ET_20_). Data are expressed as means ± SEM from 4 independent tests. Different letters show significant difference at *p* < 0.05.

Extracts of olive vegetative apexes collected from untreated (EC_8_ and EC_20_) and Se-fertilized (ET_8_ and ET_20_) plants were tested for cytosolic Ca^2+^ of control olive pollen. An aliquot (1 mg) of extract was added to the incubation medium containing CaCl_2_ (2 mM). All the extracts of the germinative apexes (EC_8_, EC_20_, ET_8_, ET_20_) determined a marked decrease in [Ca^2+^]_cp_ only for the duration of 100 s. The addition of H_2_O_2_ (0.25–5 mM) in the incubation medium, after 100 s from the reestablishment of Ca^2+^ homeostasis, determined a dose-dependent increase in [Ca^2+^]_cp_ (Figure 3). The [Ca^2+^]_cp_ was not perturbed by the hydrogen peroxide, if plant extracts of the ET_8_ or ET_20_ were present in the incubation medium (Figure 3).

Therefore, although all the extracts of the germinative apexes from untreated or Se-fertilized plants had a marked Ca^2+^ chelating activity (data not shown), the effect in [Ca^2+^]_cp_ of oxidative stress was only manifested with extracts from untreated plants (EC_8_ and EC_20_). The germinative extracts of the Se-fertilized plants (ET_8_ and ET_20_) had a total selenium content 4–5 times higher than that of the untreated plants (EC_8_ and EC_20_).

### 2.5. Germination of Olive Pollen Grains in Oxidative Stress

**Figure 4:**
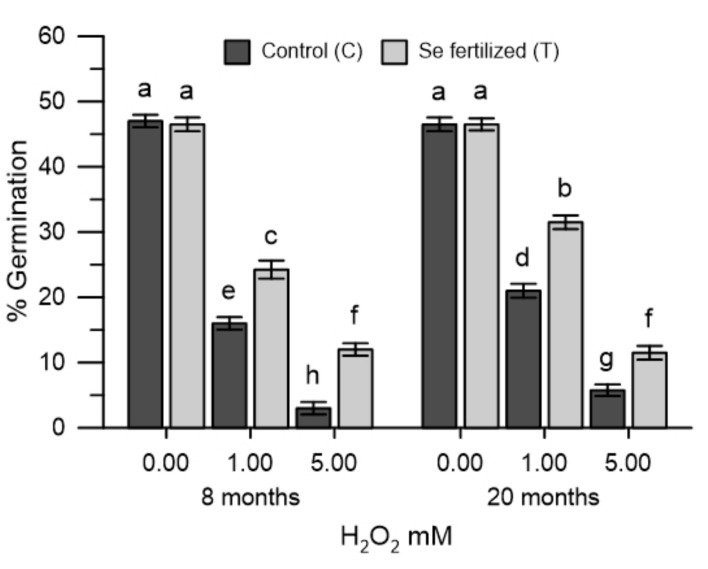
Effects of H_2_O_2_ (1 and 5 mM) in the germination of olive pollen from untreated (C_8_ and C_20_) and Se-fertilized plants (T_8_ and T_20_). Data are expressed as means ± SEM from 4 independent tests. Different letters show significant difference at *p* < 0.05.

Pollen grains collected from untreated (C_8_ and C_20_) and Se-fertilized (T_8_ and T_20_) plants were incubated for germination in the presence of H_2_O_2_ (1 mM and 5 mM). Hydrogen peroxide reduced germination differently and, respectively (Figure 4): with H_2_O_2_ (1 mM): 65% C_8_ and 46% T_8,_ 55% C_20_ and 35% T_20_; with H_2_O_2_ (5 mM): 93% C_8_ and 74% T_8_, 90% C_20_ and 76% T_20_. The results showed that the pollen grains from Se-fertilized plants are less sensitive to the effects of hydrogen peroxide in germination at both times examined and at the same concentrations of hydrogen peroxide (Figure 4).

## 3. Discussion

In this study, the determination of the half-life of Se in pollen collected 8 and 20 months after field fertilization of olive trees with Na-selenate was present in the pollen of selenium in organic (Se-met) and inorganic form, confirming what was observed in previous works [18]. Here, it emerged, for the first time, that the absorbed selenium remained in the olive pollen even after 20 months from the treatment. A modest decay was evidenced in the Se-met and in the total Se, while the inorganic species (SeVI) did not show variations over time. It is plausible that the biological self has a more dynamic metabolic turnover in olive trees. Previous studies have shown the beneficial effects of selenium in increasing the antioxidant defense against reactive oxygen species (ROS) in vegetative growth and in the response to environmental stress [14,15,16].

The determination of the variations produced in the cytosolic Ca^2+^ and in the germination were easy and quick to perform, allowing the monitoring of the oxidative stress onset and the effectiveness of the antioxidant measures proposed, as described in previous papers [25,26]. The pollen, labelled with the FURA 2AM probe, was used as an experimental model, allowing to easily trace the dynamic changes of the cytosolic Ca^2+^. In this study, oxidative stress was induced in vitro with H_2_O_2_ in olive pollen and the effects were tested in cytosolic Ca^2+^ pollen. The experimental protocol used was conducted in the absence (Ca^2+^ free) and in the presence of Ca^2+^ in the incubation medium. This allowed to differentiate, if the variations of the cytosolic Ca^2+^ caused by the oxidative stress in the pollens, resulted from the release of the ion from the internal stores or from the Ca^2+^ entry from the extracellular medium. In Ca^2+^-free conditions, H_2_O_2_ stress of the pollen internal stores caused the release of stored Ca^2+^ and the increase in cytosolic Ca^2+^. The addition of CaCl_2_ to the incubation medium resulted also in an increase in cytosolic Ca^2+^, but this was secondary to Ca^2+^ depletion. H_2_O_2_ was dependent on pollen internal stores. At both times examined, the homeostasis of Ca^2+^ in the pollen of non-treated plants in the field was altered by oxidative stress, while that of the pollen of Se-fertilized plants was not. It is presumed that the beneficial effects produced by selenium allowed the prevention of oxidative stress in pollen internal stores. A similar protective effect of selenium in cytosolic Ca^2+^, also occurred in pollens incubated with the extracts of the Se-enriched germinal apexes, collected from the same Se-fertilized olive trees. Under these experimental conditions, H_2_O_2_ did not cause changes in the levels of cytosolic Ca^2+^ and selenium also did not interfere with the Ca^2+^ -chelating properties of the extracts of the germinating apexes (data not shown). Given the short execution times of the measurement of cytosolic Ca^2+^, it is possible to exclude, at least in vitro, the implications of any metabolic mechanism and to conclude that selenium over time can substantially act as a simple ROS scavenger, which ultimately prevents the ROS-mediated dysfunction in Ca^2+^ signals. The effects of selenium were also manifested in pollen germination, which was markedly reduced by H_2_O_2_. The pollen collected after 8 and 20 months from the Se-fertilized plants showed a germination of 63–65% higher with 1 mM H_2_O_2_ than that of the pollen of untreated plants. This result is particularly important for agricultural productivity, based on multiple abiotic factors that can lead to excess ROS formation [28]. The data obtained fits in with what was asserted by some authors, who consider abiotic stresses of different nature responsible for an excessive accumulation of ROS and, consequently, for the sterility of pollen [29]. 

The SEM images of the two pollen populations did not show significant differences in size and shape, but qualitative differences emerge in the surface structure. The latter, probably, is responsible for the lower aggregation capacity of pollen. In spite of this, the Se-fertilization, despite resulting in an increase in the selenium content of about 6–8 times and of the changes in the morphology of the pollen, did not influence the germination rates in the absence of oxidative stress over time. The olive trees therefore showed an undisputed ability to adapt to selenium in the long term, thus excluding any toxicity problems.

Studies conducted previously have shown that Se-fertilization, in addition to improving the qualitative and nutritional properties of EVO oil, allowed an increase in the response of the plant to abiotic stress [14,15,16].

This study, in addition to showing the beneficial effects of selenium in cytosolic Ca^2+^ and in the germination of pollen in oxidative stress, adds, for the first time, that these effects persist over time, even after 20 months from selenium fertilization of plants and suggests the reduction in treatments in the field in order to pursue the goal of precision agriculture.

## 4. Materials and Methods

### 4.1. Reagents

FURA-2AM (FURA-2-pentakis (acetoxymethyl) ester), PBS (Phosphate-Buffered Saline), Triton X-100, EGTA (ethylene glycol-bis (β-aminoethyl ether), sodium elenite (Na_2_SeO_4_), selenium methionine, hydrogen peroxide (H_2_O_2_), sodium chloride (NaCl), potassium chloride (KCl), magnesium chloride (MgCl_2_), glucose, Hepes, and dimethyl sulfoxide (DMSO), were acquired from Sigma-Aldrich (St. Louis, MO, USA). Any other chemicals and reagents (reagent grade) were of the highest quality, and obtained from reputable commercial sources.

### 4.2. Plant Material, Growing Conditions and Pollen and Vegetative Apexes Collection

This study was conducted in 2017–2019 on trees of *Olea europaea* L., cultivar Leccino, grown in a thirty-year-old olive grove near Perugia (Central Italy, 42°57′39.2″ N, 12°25′02.5″ E). The soil is clay loam and the trees are trained to the vase system (with a trunk 1 m high and 3–4 main branches) with a planting distances of 5 × 6 m. The area has a semi-continental climate. The average temperature difference between the coldest (January) and hottest (July) months is 19–20 °C (with an average diurnal thermal range of 10–11 °C and an average annual air temperature of 13–14 °C). The maximum and minimum temperatures are 36 °C and −7 °C, respectively. The annual average precipitation is about 800 mm, distributed mostly in the autumn, winter and spring. The olive grove is considered to be representative of many intensively managed olive groves in central Italy. In the rainfed olive grove, an area away from the margins with uniform exposure, slope and chemical and physical soil characteristics was selected. Within the selected area, 20 trees (average height 3.5 m) were selected, and among them, at the end of September 10, trees with homogeneous size and yield were treated with Se (100 mg L^−1^) while another 10 trees with homogeneous size and yield similar to those treated with Se were treated with water and wetting agent only (control). Between a treated tree and a control, there were three trees that received no treatment. The Se dosage was established based on previous studies [7,10]. This solution was obtained by dissolving sodium selenate (SeO_4_^2−^) in water. For each treatment, 0.5% of the Albamilagro wetting agent (Albamilagro International S.p.A., Parabiago, MI, Italy) was added. Each plant was treated with 10 L Se solution. At the base of the tree a filter paper impermeable in the side in contact with the soil was put to prevent the solution from dripping onto the soil. On the other hand, twenty randomly selected ‘control’ trees were sprayed with the same technique, but with a water solution containing only the wetting agent. All trees reached the 1st stage of flowering in 2018 and 2019 in the last days of May. The olive phenology assessment of flowering beginning was established when the pollen was freely released by shaking the anthers of different branches, located at different heights on the tree and with different exposures [14]. At the beginning of the flowering phase, three branches for each tree (treated and control) were bagged using white double-layer paper bags (0.65 × 0.35 m) in order to collect the pollen. The bags were placed in the southeast portion of the canopy. In each tree, the bags were placed on branches of similar vigor at the apical, medial and basal positions of the part of the canopy considered. The branches had 70–80 inflorescences each. At the end of the flowering phase, the bags were removed and the pollen was filtered with a cell strainer (40 µm). At the same time from the same trees, 4 g/tree of vegetative apexes were collected. In each year (2018 and 2019), the pollen and vegetative apexes were collected from the same treated and control trees. Therefore, in summary, the pollen collection was carried out after 8 and 20 months (C_8_ and C_20_) from untreated olive trees and after 8 and 20 months (T_8_ and T_20_) from Se-fertilized olive trees.

### 4.3. Extracts of Vegetative Apexes of Olive Trees

The vegetative apexes were collected from untreated and Se-fertilized olive trees in the field after 8 (EC_8_ and ET_8_) and 20 months (EC_20_ and ET_20_) from treatment. A sample (2 g) of the collected vegetative apexes was extracted three times with 20 mL of methanol, dried and then resuspended in 10 mL of methanol. Aliquots of the extract were used to test the variations of cytosolic Ca^2+^ in olive pollen. 

### 4.4. Determination of Total Selenium in Olive Pollens and Vegetative Apexes

Measurements of total selenium content in olive pollen were performed using defrozen and dry samples, respectively. Samples of pollen (0.5 g sample^−1^) were microwave digested (ETHOS One high-performance microwave digestion system; Milestone Inc., Sorisole, Bergamo, Italy) with 8 mL of ultrapure concentrated nitric acid (65% *w*/*w*) and 2 mL of hydrogen peroxide (30% *w*/*w*). The heating program for the digestion procedure was 30 min with power of 1000 W and 200 °C. After cooling down, the digests were diluted with water up to 20 mL, then passed through 0.45 μm filters. The analysis were conducted using a graphite furnace atomic absorption spectrophotometry, Shimadzu AA-6800 apparatus (GF-AAS; GFA-EX7, Shimadzu Corp., Tokyo, Japan) with deuterium lamp background correction and a matrix modifier (Pd(NO_3_)_2_, 0.5 mol L^−1^ in HNO_3_). All analyses were carried out in triplicate.

### 4.5. Se Speciation with HPLC ICPMS

Defrozen pollen material (0.25 g) was mixed with 10 mL of solution and 2.0 mg mL^−1^ of protease. Samples were sonicated with an ultrasound probe for 2 min and stirred in a water bath at 37 °C for 4 h. Then, samples were cooled at room temperature and centrifuged for 10 min at 9000 rpm. The supernatant was filtered through 0.22 μm Millex GV filters (Millipore Corporation, Billerica, MA, USA). The standards solutions (1, 5, 10, and 20 μg L^−1^) for inorganic (i.e., selenite, SeO_3_^−2^ and selenate, SeO_4_^−2^) and organic (i.e., selenocystine, (SeCys2); Se-(methyl) selenocysteine, (SeMeSeCys); selenomethionine, (SeMet) were employed. Se forms were prepared with ultrapure (18.2 MΩ cm) water. Speciation of Se was performed by HPLC (Agilent 1100, Agilent Technologies, Santa Clara, CA, USA) using an anion exchange column (Hamilton, PRP-X100, 250 × 4.6 mm^2^, 5 μm particle size). The mobile phase was made by ammonium acetate with gradient elution. The analytical method and instrumental conditions were previously described in [9].

### 4.6. Measurement of Cytosolic Ca^2+^

Intracellular calcium levels were determined spectrofluorometrically using FURA-2AM the probe [27]. 100 mg of olive pollens were suspended in 10 mL PBS and hydrated for 3 days. Hydrated pollens were harvested by centrifugation at 1000× *g* × 4 min and then resuspended in 2 mL Ca^2+^-free HBSS buffer (120 mM NaCl, 5.0 mM KCl, MgCl2 1 mM, 5 mM glucose, 25 mM Hepes, pH 7.4). Pollen suspensions were incubated in the dark with FURA-2AM (2 µL of a 2 mM solution in DMSO) for 120 min, after which samples were centrifuged at 1000× *g* × 4 min. Pollens were then harvested and suspended in ~10 mL of Ca^2+^-free HBSS containing 0.1 mM EGTA, which was included to rule out or, at least, minimize a potential background due to contaminating ions (so as to obtain a suspension of 1 × 106 of pollen granules hydrated per mL). Oxidative stress was induced by adding hydrogen peroxide to the suspended pollen. Effects on cytosolic Ca^2+^ were evaluated after 100 s. Fluorescence was measured in a Perkin-Elmer LS 50 B spectrofluorometer (ex. 340 and 380 nm, em. 510 nm) (Figure 1), set with a 10 nm and a 7.5 nm slit width in the excitation and emission windows, respectively. Fluorometric readings were normally taken after 300–350 s. When required, samples of pollen, CaCl_2_, H_2_O_2_, Na_2_SeO_4_ and aliquot extracts of vegetative apexes were added for specific purposes, as described in the Results section. Cytosolic calcium concentrations ([Ca^2+^]c) were calculated as shown by Grynkiewicz [27].

### 4.7. Pollen Germination

Olive pollen grains used in the experimentation are collected in the field from treated trees (Se-enriched) and control trees. Freshly collected pollen samples were rehydrated by incubation in a humid chamber at room temperature for 30 min [30] and then transferred to culture plate (6-well culture plate (1.0 mg of pollen per plate) containing 3 mL of an agar-solidified growing medium: agar 1%, sucrose 10%, boric acid (H_3_BO_3_) 100 ppm and calcium chloride (CaCl_2_) 1 mM, at pH 5.5 [31]. Subsequently, with the aid of a brush, a uniform distribution was obtained on the surface of the medium. Oxidative stress was induced by adding hydrogen peroxide to the suspended pollen. Effects on germination were assessed at the end of the incubation period. Pollen grains were then incubated for 24–48 h in a growth chamber at 25 °C. The number of germinated and non-germinated pollen grains were determined with the aid of a microscope with a 10× objective lens. Germination rate were determined using two replicates of 100 grain. Grains were considered germinated if the size of the pollen tube was greater than the diameter of the grain [31]. Experiments were conducted in a completely randomized design with four replications.

### 4.8. Statistical Analysis

Statistical tests were performed using Graph Pad Prism 6.03 software for Windows (La Jolla, CA, USA). Tests for variance assumptions were conducted (homogeneity of variance by Levene’s test, normal distribution by the D’Agostino-Pearson omnibus normality test). Results obtained are expressed as mean values ± standard error of the mean (SEM). Significance of differences were analyzed by Fisher’s least significant differences test, after the analysis of variance according to the split plot in time design. Differences with *p* < 0.05 were considered statistically significant.

## 5. Conclusions

This study shows that through the determination of cytosolic Ca^2+^ and pollen germination, quick and easy measurements are possible to monitor the onset of oxidative stress and the effectiveness of any antioxidant measure adopted. Furthermore, the field treatment with selenium maintains its effects even after 20 months, suggesting that it is possible to reduce the amount of selenium fertilization in a precision agriculture perspective.

## Figures and Tables

**Table 1:** Speciation of Se in olive pollen grains collected after 8 and 20 months from untreated (C_8_ and C_20_) and Se-fertilized (T_8_ and T_20_) olive trees in the field.

	MeSeCys ppb	Se Met ppb	Se (IV) ppb	Se (VI) ppb	Se Total ppb
C_8_	23 ± 3 a	858.2 ± 15 a	300.5 ± 8 a	1725.0 ± 32 a	3210 ± 30 a
C_20_	41 ± 4 a	778.7 ± 14 a	460.7 ± 9 b	1883.2 ± 33 a	3600 ± 29 a
T_8_	153 ± 10 b	4895.2 ± 23 c	218.4 ± 7 a	15,860.0 ± 34 b	28,370 ± 51 b
T_20_	211.8 ± 12 b	2541.2 ± 22 b	582.4 ± 11 b	14,563.0 ± 31 b	17,900 ± 41 b

Means in each column followed by the different letter are significantly different at *p* < 0.05.

## Data Availability

All raw or derived data supporting the findings of this study are available from the corresponding author, upon request.
